# The iNOS Activity During an Immune Response Controls the CNS Pathology in Experimental Autoimmune Encephalomyelitis

**DOI:** 10.3389/fimmu.2019.00710

**Published:** 2019-04-04

**Authors:** Sandip Ashok Sonar, Girdhari Lal

**Affiliations:** National Center for Cell Science, Pune, India

**Keywords:** experimental autoimmune encephalomyelitis, inducible nitric oxide synthase, NOS2^−/−^ neuroinflammation, central nervous system, autoimmunity

## Abstract

Inducible nitric oxide synthase (iNOS) plays a critical role in the regulation of multiple sclerosis (MS) and experimental autoimmune encephalomyelitis (EAE). Previous studies have shown that iNOS plays pathogenic as well as regulatory roles in MS and EAE. However, how does iNOS alters the pathophysiology of the central nervous system (CNS) in neuronal autoimmunity is not clearly understood. In the present work, we show that treatment of mice with L-NAME, an iNOS inhibitor, during the antigen-priming phase primarily alters brain pathology, while in the subsequent effector phase of the immune response, the spinal cord is involved. Inhibition of iNOS during the priming phase of the immune response promotes the infiltration of pathogenic CD11b^+^F4/80^−^Gr-1^+^ cells, but there is low recruitment of regulatory CD11b^+^F4/80^+^ cells in the brain. Inhibition of iNOS during the effector phase shows similar pathogenic alterations in the spinal cord, instead of in the brain. Treatment of wild-type mice with L-NAME or mice having genetic deficiency of iNOS show lower MHC-II expression on the dendritic cells, but not on macrophages. Our data suggest that iNOS has a critical regulatory role during antigen-priming as well as in the effector phase of EAE, and inhibition iNOS at different stages of the immune response can differentially alter either the brain or spinal cord pathology. Understanding the cellular and molecular mechanisms through which iNOS functions could help to design a better strategies for the clinical management of neuroinflammation and neuronal autoimmunity.

## Introduction

Nitric oxide (NO) is a small bioactive lipophilic molecule that diffuse across the cell membrane and controls many physiological functions of the body (1). NO production requires nitric oxide synthase (NOS), which catalyze the oxidation of L-arginine to L-citrulline (2, 3). In mammals, there are three different isoforms of NOS - endothelial NOS (eNOS), neuronal NOS (nNOS) and inducible NOS (iNOS) (4). Constitutive expression of eNOS and nNOS controls the vasodilation of vessels and neuronal functions, respectively (5). Several inflammatory stimuli can induce the expression of iNOS in various cell types such as macrophages, dendritic cells, neutrophils, epithelial cells in the gut and lung mucosa, smooth muscle cells, and stromal cells of secondary

lymphoid organs (6–9). iNOS is also expressed in microglial cells, astrocytes, neurons in the central nervous system (CNS), and endothelial cells at the blood-brain barrier (BBB) (7, 10, 11). A clinical association between iNOS and pathogenesis has been reported in many organ-specific autoimmune inflammatory diseases, including multiple sclerosis (MS) and experimental autoimmune encephalomyelitis (EAE) (12–15).

Proper neuronal function requires the presence of the minimal physiological concentrations of NO in the CNS, with sustained high NO levels leading to detrimental effects (16). The active MS patients show high NO levels in the cerebrospinal fluid (CSF), and high concentrations of NO, peroxynitrite, and other reactive nitrogen species have been found to correlate with greater severity and chances of relapse of clinical symptoms (15, 17). The expression of iNOS in the CNS is very tightly regulated, and several intrinsic and extrinsic stimuli can induce its expression in immune cells (14, 18). The T cell-derived cytokine, IFN-γ induces the expression of iNOS in macrophages and microglial cells which leads to the generation of higher NO and peroxynitrite productions, and cause tissue destruction in the CNS (13, 19, 20). However, iNOS^−/−^ mice are hypersusceptible to EAE, suggesting that iNOS may have a regulatory function during CNS inflammation and autoimmunity (21, 22). Several studies have shown that iNOS can regulate the function of regulatory dendritic cells (regulatory DCs) which in turn can induce apoptosis of inflammatory CD4^+^ T cells and help in controlling the development of EAE (23–25). Furthermore, iNOS expression in macrophages is linked with the suppression of inflammasome activation-induced IL-1β production (26), as well as a reduction in the frequency of M1 macrophages (27). Myeloid cell-derived iNOS is also known to control the CD4^+^ T cell response (28, 29). High levels of APCs-derived NO suppresses CD4^+^ T cell response, while low levels favor the generation of a Th1 response (30). Th17-intrinsic iNOS has been shown to suppress Th17 response through nitration of the tyrosine residues of RORγt, and limiting its promoter binding capacity (31). While it is known that Th17 cells in the CNS express iNOS during EAE, the functional importance of iNOS production by Th17 cells in the inflamed CNS is not clearly understood. During chronic demyelination, a pathogenic phenotype of microglial cells has been found to be associated with iNOS expression (32, 33). Since both lesion-associated and non-associated astrocytes express iNOS, the contribution of astrocyte-derived iNOS is still unclear. Some *in vitro* experiments suggest that inflammatory cytokine-induced iNOS reduces the expression of myelin proteins and causes oligodendrocyte death in the mixed glial cultures (34). All these observations indicate that iNOS plays a dual role during neuronal autoimmunity. Anti-IFN-γ treatment and IFN-γR^−/−^ mice show hypersusceptibility to the development of EAE, with preferential involvement of the brain stem and cerebellum, resulting in the atypical EAE symptoms with the critical participation of neutrophil effector function (35–37). Given that IFN-γ regulates the iNOS expression in several immune cells, how does iNOS controls the inflammation in the brain and the spinal cord, and whether iNOS performs different functions during the antigen-priming and effector phases of EAE is not known.

In the present study, we assessed the role of iNOS using L-NAME-mediated inhibition of its activity during various stages of the immune response in EAE, including the antigen-priming phase and the effector phases, accompanied by monitoring of cellular pathology in the CNS. Our results showed that inhibition of iNOS during the antigen-priming as well as effector phases of EAE worsened the disease, and histology indicated differential regulation of infiltration of CD11b^+^F4/80^−^GR-1^+^ and CD11b^+^F4/80^+^ cells in the brain and spinal cord. iNOS inhibition during the antigen-priming phase selectively promoted the infiltration of inflammatory CD11b^+^F4/80^−^GR-1^+^ cells, while lowering the frequency of infiltration of CD11b^+^F4/80^+^ cells into the brain. Conversely, inhibiting iNOS during the effector phase led to mostly CD11b^+^F4/80^−^GR-1^+^ cells migrating into the spinal cord. A similar phenotype with higher infiltration of CD11b^+^F4/80^−^GR-1^+^ cells and reduced infiltration of CD11b^+^F4/80^+^ cells in the CNS was observed in iNOS^−/−^ mice or wild-type mice in which IFN-γ, a known inducer of iNOS, was blocked. We show that iNOS plays a regulatory role in promoting the infiltration of CD11b^+^F4/80^+^ suppressor cells, while at the same time inhibiting the mobilization of pathogenic CD11b^+^F4/80^−^GR-1^+^ cells into the CNS.

## Results

### Inhibition of NO Production in the Priming Phase Promotes Granulocytic Myeloid Cells Infiltration Specifically in the Brain

**Figure 1:**
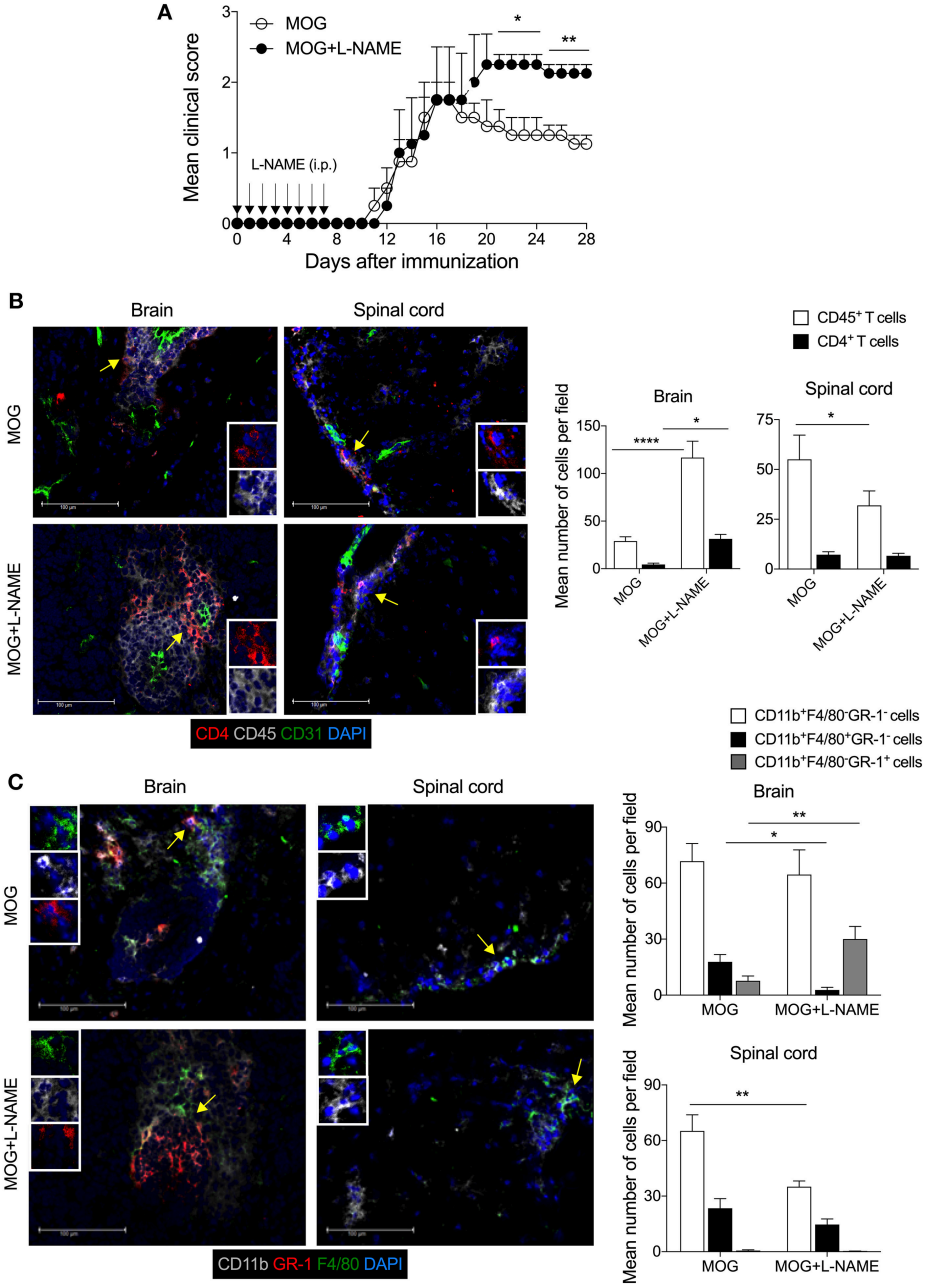
Inhibition of iNOS during the antigen-priming phase of EAE lead to increased infiltration of myeloid cells in the brain. C57BL/6 mice were *s.c*. injected with 200 μg MOG_35−55_ (MOG) in CFA emulsion, and two doses of *i.v*. pertussis toxin (PTx, 200 ng/mouse) at day 0 and 2. Mice were administered *i.p*. with L-NAME (100 mg/kg) from day 0 to 7 daily. Control groups were given i.p. PBS. **(A)** EAE clinical score was monitored and plotted. Arrow shows the day of L-NAME injection. Error bars represent ± standard error of mean (SEM). **(B)** Mice were sacrificed on day 28, and the brain and spinal cord were analyzed by immunofluorescence staining. Representative images of CD4 (red), CD45 (gray), CD31 (green), and nuclear stain DAPI (blue) stained brain and spinal cord tissues are shown (left). The mean number of infiltrated CD4^+^ T cells and CD45^+^ leukocytes were quantified from at least 25–30 sections of the brain and spinal cord and plotted (right). **(C)** Representative images of the brain and spinal cord tissues stained with CD11b (gray), GR-1 (red). F4/80 (green), and nuclear stain DAPI (blue) are shown (left). The mean number of infiltrated CD11b^+^F4/80^−^GR-1^−^, CD11b^+^F4/80^+^GR-1^−^ and CD11b^+^F4/80^−^GR-1^+^ cells from at least 25–30 sections of the brain and spinal cord are plotted (right). Error bars represent ± standard error of mean (SEM) **(A–C)**. Original magnification 400x **(B,C)**. Scale bar 100 μm **(B,C)**. ^*^p < 0.05, ^**^p < 0.01, ^****^p < 0.0001; two way ANOVA followed by Tukey's test **(B)**, Student *t*-test **(A,C)**. *n* = 5 mice/group.

Active EAE was induced in C57BL/6 mice and given an intraperitoneal injection of NOS inhibitor L-NAME (100 mg/kg/ every day) in the antigen-priming phase (one injection/day for seven days). The inhibition of NO production by L-NAME at the antigen-priming phase significantly increased the severity of EAE mice, compared to control mice (Figure 1A). Interestingly, the severity of the clinical symptoms of EAE increased with time in mice that received L-NAME (Figure 1A). Analysis of the brain and spinal cord tissues showed enhanced infiltration of CD45^+^ leukocytes, including CD4^+^ T cells, in the brain but not in the spinal cord (Figure 1B). CD11b^+^F4/80^+^ cells include mainly macrophages and monocytic myeloid-derived suppressor cells (Mo-MDSCs). The F4/80-expressing MDSCs are known to have a suppressive function in EAE (38), whereas CD11b^+^GR-1^+^ cells show a pathogenic phenotype (39). L-NAME-treated mice showed a significant reduction in the infiltration of CD11b^+^F4/80^+^GR-1^−^ cells selectively in the brain, but not in the spinal cord (Figure 1C). However, L-NAME treatment significantly increased infiltration of CD11b^+^F4/80^−^GR-1^+^ cells (mainly neutrophils) in the brain but not in the spinal cord (Figure 1C). Together, our results showed that inhibition of NOS in the priming phase of EAE mostly affected the infiltration of inflammatory CD4^+^ T cells and GR-1^+^ neutrophils in the brain, and reduced the frequency of CD11b^+^F4/80^+^ cells, which might account for the severity of EAE in the later phase of the disease.

### Inhibition or Deficiency of iNOS in the Antigen-Priming Phase Does Not Alter the Differentiation of Effector CD4^+^ T Cells

**Figure 2:**
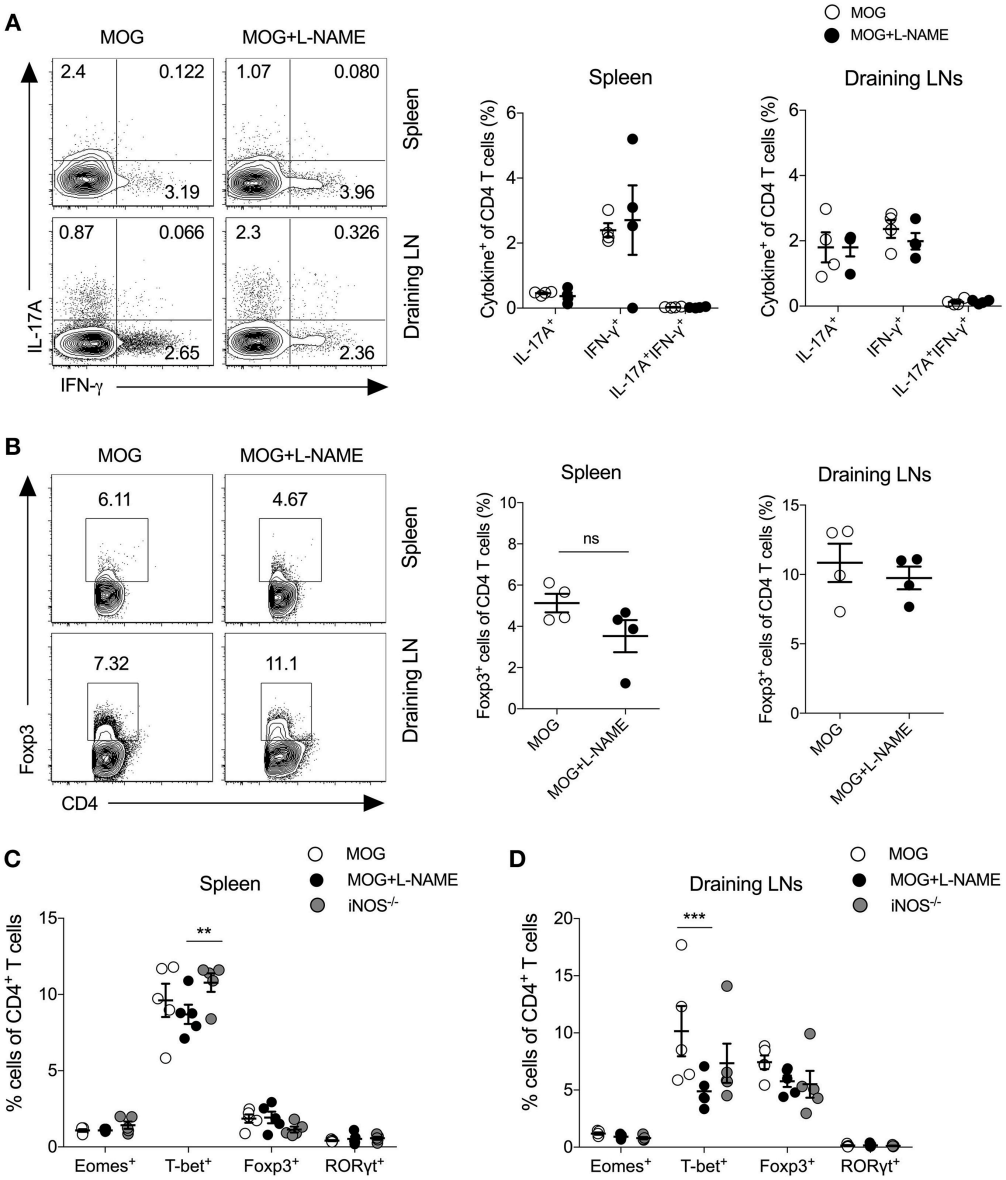
Inhibition of iNOS during the antigen-priming phase of EAE does not affect Th1, Th17, and Tregs in the secondary lymphoid organs. Active EAE was induced as given in Figure 1. **(A,B)** On day 28, single cell suspensions were prepared from the spleen and draining lymph nodes. Intracellular IL-17A and IFN-γ were analyzed in the CD4^+^ T cells using flow cytometry and plotted. **(A)** FACS plots show the intracellular expression of IL-17A and IFN-γ in the CD4^+^ T cells (left). Quantifications of the percentage of intracellular cytokines in the CD4^+^ T cells are shown (right). **(B)** Foxp3 expression in the CD4^+^ T cells (left). Quantifications of Foxp3^+^CD4^+^ T cells (right). **(C,D)** C57BL/6 and iNOS^−/−^mice were *s.c*. injected with 200 μg MOG_35−55_ (MOG) in CFA emulsion, and two doses of *i.v*. pertussis toxin (PTx, 200 ng/mouse) at day 0 and 2. Mice were administered *i.p*. L-NAME (100 mg/kg) from day 0 to 7 daily. Control groups were given *i.p*. PBS. On day 8, single cell suspensions were prepared, and expression of Eomes, T-bet, Foxp3, and RORγt were analyzed in CD4^+^ T cells. **(C)** Spleen and **(D)** draining lymph nodes were analyzed using flow cytometry and plotted. Numbers in the dot plots show the percentages of CD4^+^ T cells **(A,B)**. Each dot represents an individual mouse, and the horizontal line denotes mean and error bars represent ± SEM **(A–D). (A–D)**. Student's *t*-test **(A,B)**, ^**^p < 0.01, ^***^p < 0.001; ANOVA followed by Tukey's test **(C,D)**. *n* = 4–5 mice/group.

Since L-NAME injections at day 0-7 coincided with the antigen-specific priming, activation, and differentiation of effector CD4^+^ T cells, we measured the frequency of Th1 cytokine (IFN-γ) and Th17 cytokine (IL-17A) producing cells, and Foxp3^+^ regulatory CD4^+^ T cells in the spleen and lymph nodes. Our results showed no significant alteration in the intracellular expression of IL-17A and IFN-γ in CD4^+^ T cells (Figures 2A,B) or γδ T cells (Figure S1A). To further confirm the role of iNOS in the priming-phase of EAE, we immunized wild-type and iNOS^−/−^ mice with MOG in complete Freund's adjuvant (CFA). On day 8, we compared the differentiation of effector immune cells in their spleen and lymph nodes with those of L-NAME (d0-7)-treated and untreated wild-type C57BL/6 mice. iNOS^−/−^ mice also did not show any significant change in the expression of the Th lineage-specific transcription factors, T-bet, RORγt, Foxp3, and Eomes in CD4^+^ T cells, as compared to the control group (Figures 2C,D and Figures S1B,C). However, L-NAME treated mice showed a lower frequency of T-bet expressing CD4^+^ T cells in the draining lymph nodes, as compared to the control group (Figures 2C,D). We did not observe any significant changes in the expression of intracellular IL-17A, IFN-γ, and GM-CSF in the CD4^+^ T cells or γδ T cells (data not shown). On day 28, L-NAME treated mice showed severe EAE symptoms but did not show a significant change in the percentages of Th1, Th17, and Tregs or IL-17A^+^ and IFN-γ^+^ or IL-17A^+^IFN-γ^+^ γδ T cells in the secondary lymphoid organs (Figures 2A,B and Figure S1A). Together, these results suggest that inhibition of iNOS during the priming phase of EAE does not affect the differentiation of Th1, Th17, and Treg cells in the secondary lymphoid tissues.

### Inhibition of iNOS in the Effector Phase Shows Increase Cellular Infiltration in the Spinal Cord

**Figure 3:**
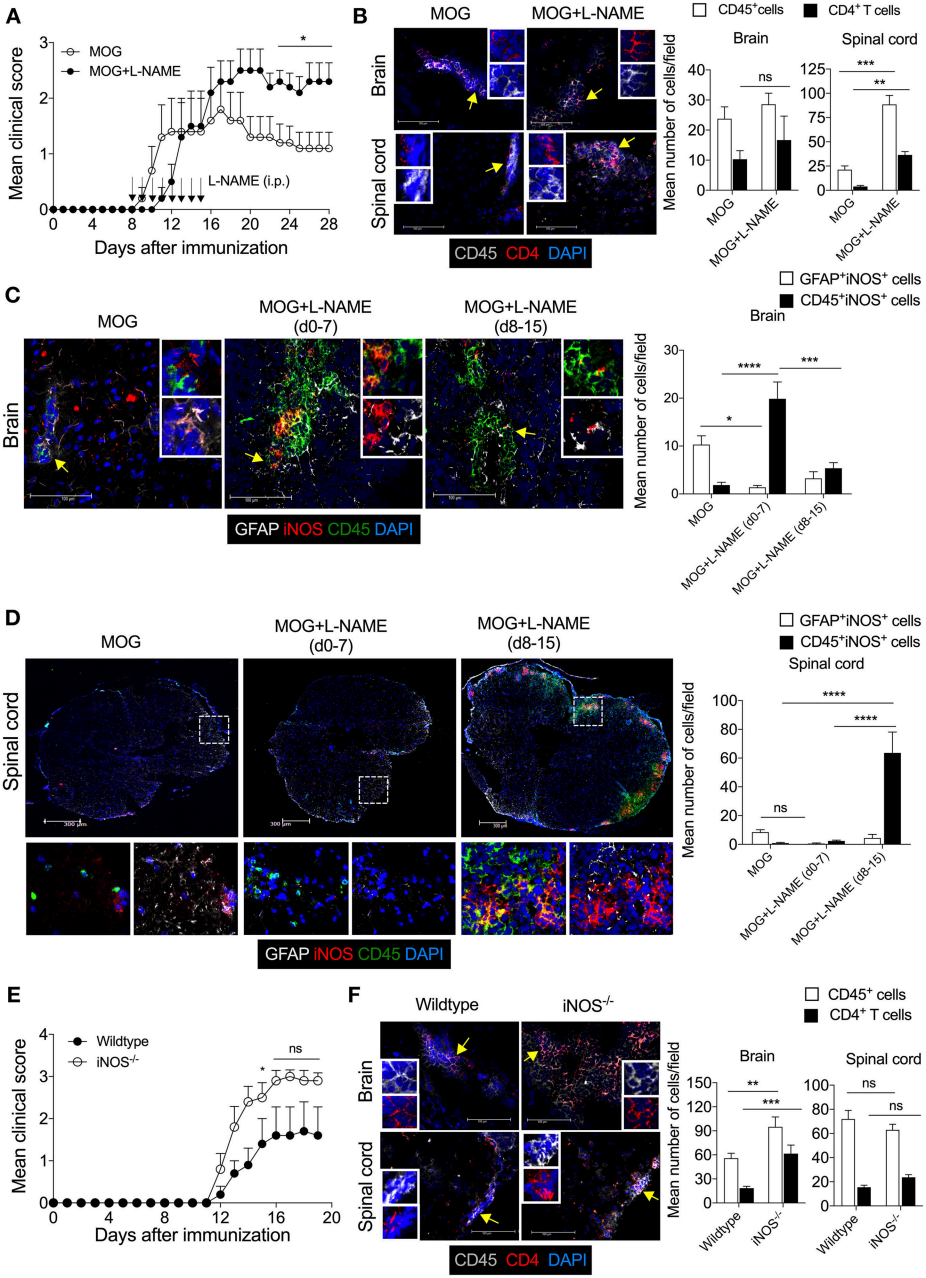
The Inhibition of iNOS during the effector phase exacerbates inflammation in the spinal cord, leading to the development of severe EAE. Active EAE was induced as in Figure 1. Mice were administered *i.p*. L-NAME from day 8 to 15 daily. Control groups were given *i.p*. PBS. **(A)** EAE clinical scores were monitored and plotted. **(B)** On day 28, brain and spinal cord cryo-sections were analyzed by immunofluorescence staining. Representative images of the brain and spinal cord tissue sections stained with CD45 (gray), CD4 (red), and nuclear stain DAPI (dark blue) are shown (left). Mean numbers of infiltrating CD45^+^ and CD4^+^ cells from 16 to 25 images of the brain and spinal cord were quantitated and plotted (right). **(C,D)** Representative images of the **(C)** brain and **(D)** spinal cord sections stained with GFAP (gray), iNOS (red), CD45 (green), and nuclear stain DAPI (dark blue) are shown (left). Magnified images of the regions marked with the dotted square and are shown at the bottom **(D)**. Mean numbers of GFAP^+^iNOS^+^ and CD45^+^iNOS^+^ cells from 15 to 35 images of the brain and spinal cord were quantitated and plotted (right). **(E)** C57BL/6 and iNOS^−/−^ mice were *s.c*. injected with 200 μg MOG_35−55_ (MOG) in CFA emulsion, and two doses of *i.v*. pertussis toxin (PTx, 200 ng/mouse) at day 0 and 2. Mice were monitored for the development of clinical symptoms of EAE and the mean clinical scores were plotted (5 mice/group). **(F)** Mice in **(E)** were sacrificed on day 20, and brain and spinal cord cryo-sections were analyzed by immunofluorescence staining. Representative images of the brain and spinal cord tissue sections stained with CD45 (gray), CD4 (red), and nuclear stain DAPI (dark blue) and analyzed are shown (left). Mean numbers of infiltrating CD45^+^ and CD4^+^ cells from 25 to 35 images of the brain and spinal cord were quantitated and plotted (right). Error bars represent ± SEM **(A–F)**. Original magnification 400x (Brain; **B,C,F**); 200x (spinal cord; **B,D,F**). Scale bar, 100 μm (Brain; **B,C,F**), 300 μm (spinal cord; **B,D,F**). ^*^p < 0.05, ^**^p < 0.01, ^***^p < 0.001, ^****^p < 0.0001; Student's *t-*test **(A,B,F)**, two way ANOVA followed by Tukey's test **(C,D)**. *n* = 5 mice/group.

To understand how inhibition of NO production during the effector phase of EAE changes the pathophysiological phenotype and transmigration of effector immune cells in the CNS, L-NAME was administered (100 mg/kg/day; i.p.; day 8–15 of MOG injection) in C57BL/6 mice. Inhibition of NO production during the effector phase resulted in exacerbated EAE in these mice, as compared to control mice (Figure 3A). Interestingly, while increased infiltration of CD45^+^ leukocytes and CD4^+^ T cells was observed in the spinal cord in mice treated with L-NAME in the effector phase, infiltration of these cells in the brain was similar in L-NAME-treated and control mice (Figure 3B). This suggested that inhibition of NO production during the effector phase of EAE differentially diverts the inflammatory cells to the spinal cord and worsens the disease. Both meningeal and parenchymal regions showed higher infiltration in L-NAME-treated mice, as compared to control group (data not shown). Surprisingly, immunohistological analysis showed higher expression of iNOS in the brain-infiltrating CD45^+^ leukocytes in the priming phase, as compared to that observed upon treatment with L-NAME in the effector phase (Figure 3C). However, inhibition of NO production during the effector phase showed a higher frequency of iNOS-expressing CD45^+^ leukocytes in the spinal cord as compared to control group, or the mice that received iNOS inhibitor in the priming phase (Figure 3D). Analysis of iNOS expression in the brain and spinal cord astrocytes further revealed that the frequency of iNOS-expressing GFAP^+^ astrocytes was significantly lower in the brains of mice treated with L-NAME during the antigen-priming phase as compared to control, whereas the spinal cord resident astrocytes did not show alteration in the number of iNOS-expressing cells (Figures 3C,D). These results indicate that L-NAME-mediated transient inhibition of NOS during the antigen-priming and effector phase of EAE leads to the reappearance of iNOS expression selectively in the infiltrated CD45^+^ leukocytes and possibly in the CD45^int^ CNS-resident microglial cells, in the brain and spinal cord, respectively. Consistent with the previous reports (21, 22), our results also showed that the a genetic deficiency of iNOS in mice leads to the development of more severe EAE than that observed in wild-type animals (Figure 3E). Immunohistological analysis of the brain and spinal cord in iNOS^−/−^ mice showed significantly increased infiltration of CD45^+^ leukocytes and CD4^+^ T cells in the brain but not in the spinal cord (Figure 3F). These observations were consistent with those we obtained with the L-NAME injections in the early priming phase of the EAE, suggesting that lack of NO production in the initial stage of EAE directs pathological mechanisms specifically to the brain but not the spinal cord.

Furthermore, inhibition of NO production during the effector phase showed significantly reduced infiltration of CD11b^+^F4/80^+^GR1^−^ cells in the brain and spinal cord as compared to control animals (Figure S2A). Interestingly, substantially higher infiltration of CD11b^+^F4/80^−^GR-1^+^ cells was observed in the spinal cord but not in the brain tissue during effector phase iNOS inhibition as compared to control mice (Figure S2A).

To further confirm the role of iNOS in the infiltration of the CD11b^+^F4/80^+^GR-1^−^ cells and CD11b^+^F4/80^−^GR-1^+^ cells in the CNS, we induced active EAE in iNOS^−/−^ mice and analyzed myeloid cell infiltration in the brain and spinal cord. Consistent with our observations with L-NAME treatment during the priming or effector phase, our results showed that deficiency of iNOS leads to reduced infiltration of CD11b^+^F4/80^+^GR-1^−^ cells and increased infiltration of CD11b^+^F4/80^−^GR-1^+^ cells in the brain and spinal cord, as compared to wild-type mice (Figure S2B). These results suggest that inhibition of NOS or a genetic deficiency of iNOS selectively promotes the neutrophilic infiltration and inhibits the infiltration of CD11b^+^F4/80^+^GR-1^−^ cells in the CNS.

Together, our results show that lack of iNOS during EAE development facilitates inflammation in both the brain and spinal cord, with enhanced inflammatory cell infiltrations specifically in the brain but not the spinal cord as compared to wild-type mice. However, differential preference observed with L-NAME-mediated inhibition of NOS during the priming versus effector phase of EAE. These results suggest that inhibition of NOS in the different stages of EAE differentially regulates the infiltration of effector CD45^+^ leukocytes, CD4^+^ T cells, CD11b^+^F4/80^−^GR-1^+^ cells, and CD11b^+^F4/80^+^GR1^−^ cells in the CNS, and this may, in turn, account for the severity of the disease.

### Inhibition of iNOS During the Priming-Phase Reduces MHC Class II Expression on DCs and Reduces the Frequency of Monocytic-MDSCs in Secondary Lymphoid Tissues.

**Figure 4:**
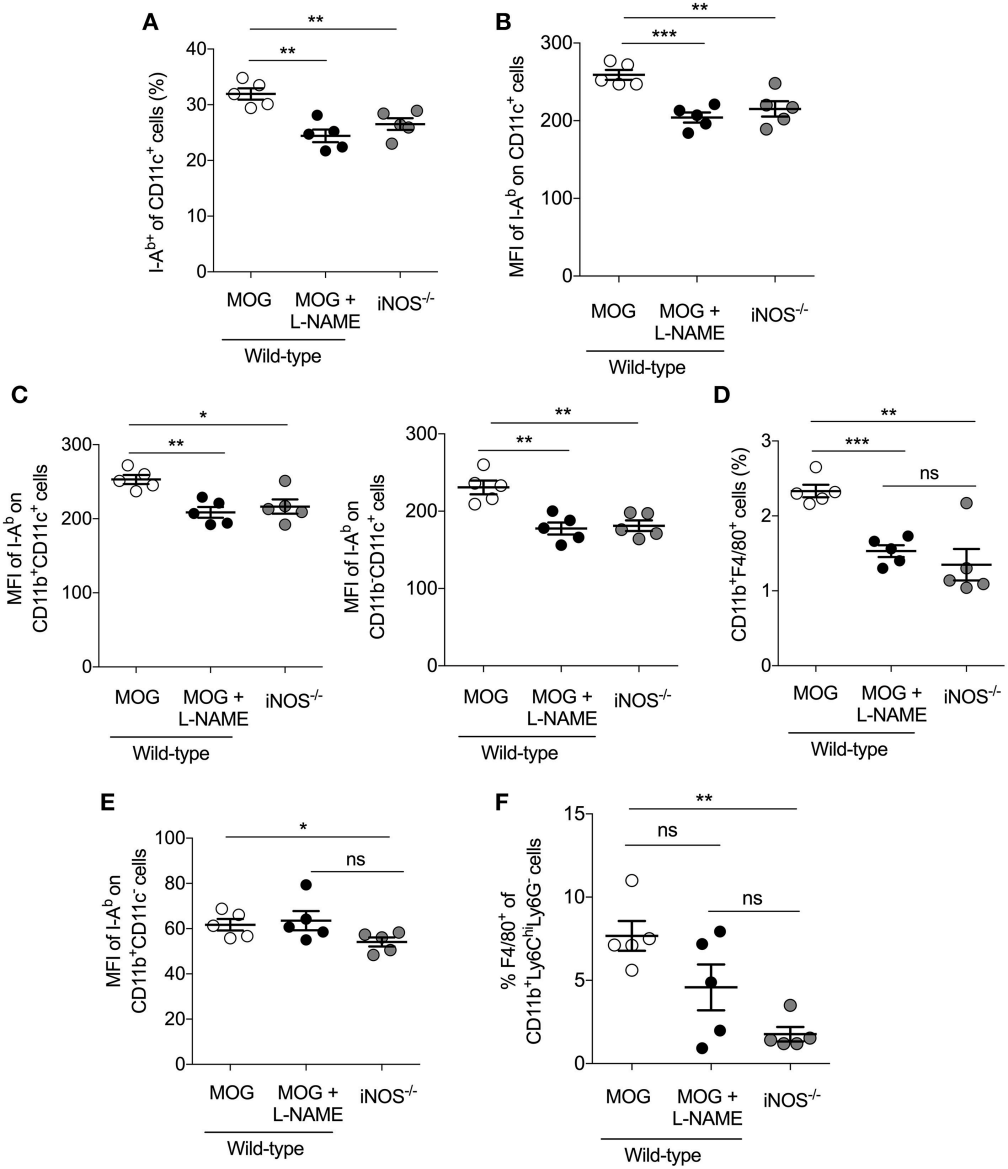
Inhibition of iNOS in the priming phase inhibits MHC class II expression on DCs and reduces the frequency of monocytic-MDSCs in the secondary lymphoid tissues. C57BL/6 and iNOS^−/−^mice were *s.c*. injected with 200 μg MOG_35−55_ (MOG) in CFA emulsion, and two doses of *i.v*. pertussis toxin (PTx, 200 ng/mouse) at day 0 and 2. Mice were administered *i.p*. with L-NAME (100 mg/kg) from day 0 to 7 daily. Control groups were given *i.p*. PBS. On day 8, single cell suspensions were prepared, and myeloid cell populations in the secondary lymphoid organs were analyzed using flow cytometry. **(A)** The percentages of I-A^b+^CD11c^+^ cells and **(B)** mean fluorescence intensity (MFI) of I-A^b^ expression on CD11c^+^ cells are shown **(C)** The MFI of I-A^b^ on CD11b^+^CD11c^+^, CD11b^−^CD11c${}_{,}^{+}$ and CD11b^−^CD11c^+^ myeloid cells were quantitated and plotted. **(D)** Data show the percentage of CD11b^+^F4/80^+^ cells in the spleen. **(E)** The MFI of I-A^b^ on CD11b^+^CD11c^−^ cells were analyzed and plotted. **(F)** The percentage of F4/80^+^ of CD11b^+^Ly6C^hi^Ly6G^−^ cells in the spleen were analyzed and plotted. Each dot represents an individual mouse, and the horizontal line denotes mean and error bars represent ± SEM. **(A–F)**. Student's *t*-test **(A–F)**. ^*^p < 0.05, ^**^p < 0.01, ^***^p < 0.001; *n* = 5 mice/group.

Myeloid cells like macrophages and dendritic cells serve as antigen presenting cells (APCs) and significant producers of iNOS, and play a crucial role in the pathogenesis of EAE (23, 24). Our result showed that EAE induced in iNOS^−/−^ mice, as well as L-NAME-treated mice (day 0–7), had significantly reduced frequency of CD11c^+^I-A^b+^ DCs (Figure 4A), and significantly lower median fluorescence intensity (MFI) of the class II MHC molecule (I-A^b^) on the DCs, as compared to control mice (Figure 4B). Further analysis showed that among the various subsets of DCs, the CD11b^+^CD11c^+^ and CD11b^−^CD11c^+^ subsets showed reduced expression of class II MHC in L-NAME-treated mice and iNOS^−/−^ mice, as compared to control EAE mice (Figure 4C). L-NAME-treated and iNOS^−/−^ mice also showed a reduced percentage of CD11b^+^F4/80^+^ macrophages in the spleen (Figure 4D), which may correspond to the lower infiltration of CD11b^+^F4/80^+^ cells observed in the brain of L-NAME-treated mice, as compared to untreated mice (Figure 1C and Figure S2B). L-NAME treatment did not have any effect on the MFI of I-A^b^ on non-DCs, whereas a lack of iNOS in iNOS^−/−^ mice showed a significant decrease in the MFI of I-A^b^ on non-DCs, as compared to control mice (Figure 4E). The F4/80^+^CD11b^+^Ly6C^hi^Ly6G^−^ cells mainly represent Mo-MDSCs, and are potent suppressors of effector CD4^+^ and CD8^+^ T cells, and known to express their suppressive effect via an iNOS-dependent mechanism (38). Our results showed a significantly reduced frequency of F4/80-expressing Mo-MDSCs in the iNOS^−/−^ mice spleen, and to some extent in the L-NAME-treated mice, as compared to control mice (Figure 4F). These results suggest that inhibition or lack of iNOS can alter the generation of CD11b^+^F4/80^+^ cells, including suppressive F4/80^+^Mo-MDSCs and mature antigen-presenting DCs in the spleen, leading to reduced infiltration of CD11b^+^F4/80^+^GR-1^−^ cells and increased infiltration of CD11b^+^F4/80^−^GR-1^+^ cells in the brain.

### Neutralization of IFN-γ Shows iNOS Expression and Apoptosis of CNPase^+^ Oligodendrocytes in the CNS

IFN-γ is a potent inducer of class II MHC molecules on APCs. It can render either inflammatory or tolerogenic function to DCs, depending on the presence or absence of a Toll-like receptor (TLR) and CD40L signaling, and is essential for the development of allograft tolerance. IFN-γ is also a known inducer of iNOS in a variety of cell types, including neutrophils, monocytes, macrophages, dendritic cells, microglial cells, and astrocytes (28). The IFN-γ^−/−^ or IFN-γR^−/−^ mice or antibody-mediated neutralization of IFN-γ signaling, confers hyper-susceptibility to the EAE. Similarly, our findings revealed that L-NAME-mediated inhibition of NOS in the early and effector phase of EAE causes severe EAE in wild-type mice.

**Figure 5:**
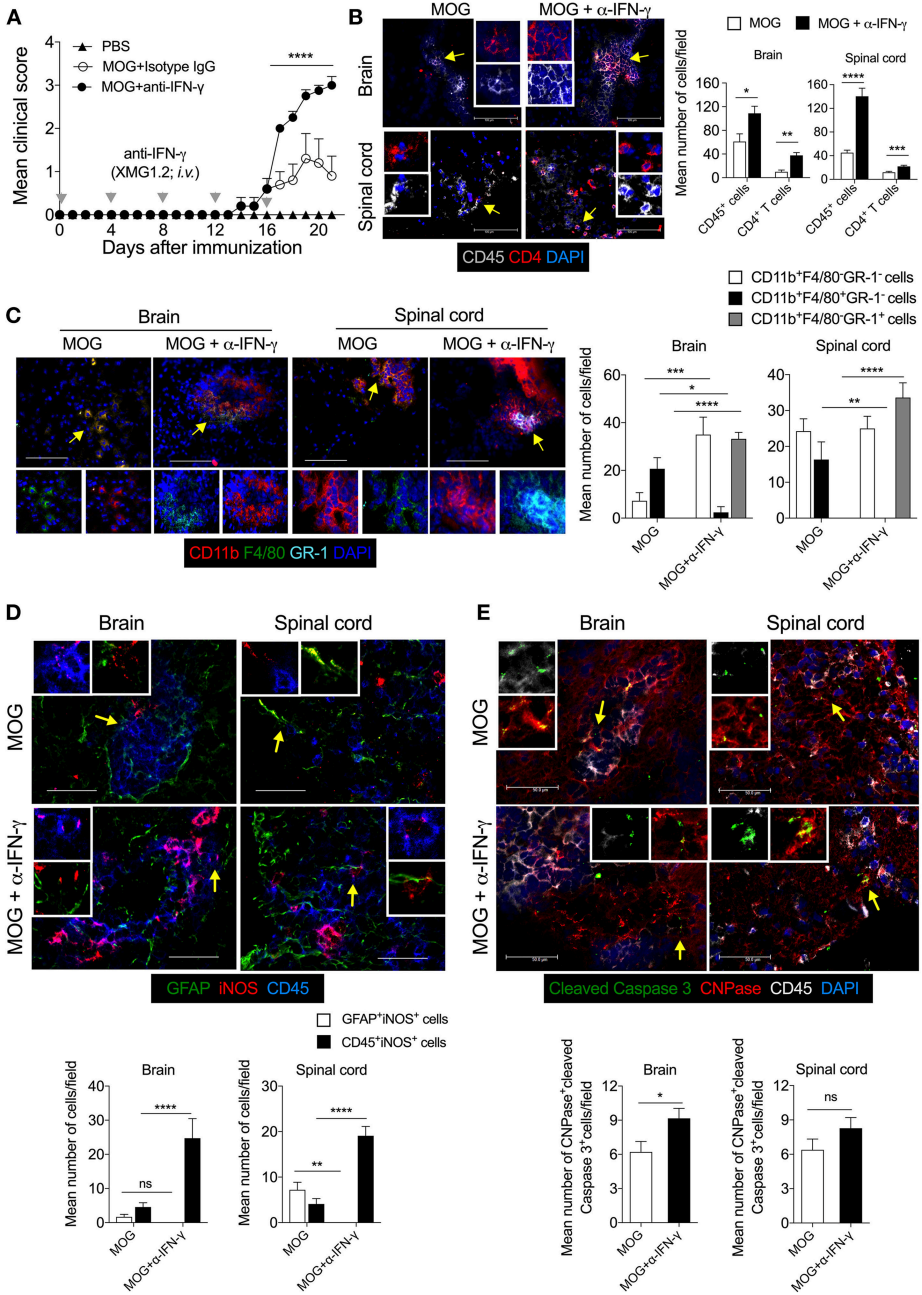
Neutralization of IFN-γ induces the expression of iNOS in the effector immune cells and results in apoptosis of CNPase^+^ oligodendrocytes in EAE. Active EAE was induced in C57BL/6 mice. Anti-IFN-γ monoclonal antibody (XMG1.2, 100 μg/mouse) monoclonal antibody was *i.v*. injected at days 0, 4, 8, 12, 16 after MOG-immunization. **(A)** The clinical symptoms of EAE were recorded and mean clinical scores are plotted (5 mice/group). **(B)** On day 21, the brain and spinal cord cryo-sections were analyzed by immunofluorescence staining. Representative images of the brain and spinal cord tissue sections stained with CD45 (gray), CD4 (red), and nuclear stain DAPI (dark blue) are shown (left). Mean numbers of infiltrating CD45^+^ and CD4^+^ cells from 25 to 35 images of the brain and spinal cord were quantitated and plotted (right). **(C)** Representative images of the brain and spinal cord stained with CD11b (red), F4/80 (green), GR-1 (light blue), and nuclear stain DAPI (dark blue) are shown (left). Infiltration by indicated cell populations was quantified and plotted (right). **(D)** Representative images stained with GFAP (green), iNOS (red) and CD45 (blue) are shown (left). iNOS-expressing GFAF^+^ cells and CD45^+^ cells were quantified from 20 to 30 images of the brain and spinal cord and plotted as mean number of cells/field (right). **(E)** Representative images stained for cleaved caspase 3 (green), CNPase (red), CD45 (gray), and DAPI (blue) are shown (left). Cleaved-caspase 3 expressing CNPase^+^ cells oligodendrocytes were quantified from 20 to 30 images of the brain and spinal cord and plotted as mean number of cells/field (right). Regions marked with white dotted square **(D,E)** are shown as magnified images at the right. Error bars represent ± SEM **(A–E)**. Original magnification 200x (**B**, spinal cord), 400x (**B**, brain**)** and **(C)**, 630x **(D,E)**. Scale bar 50 μm **(D,E)**, 100 μm (**B**, brain) and **(C)** and 300 μm (**B**, spinal cord). ^*^p < 0.05, ^**^p < 0.01, ^***^p < 0.001, ^****^p < 0.0001, ANOVA followed by Tukey's test **(A)**, ANOVA followed by Sidak's test **(C)**, Student's *t*-test **(B,D,E)**. *n* = 5 mice/group.

Since our results showed that lack of iNOS reduced the expression of class II MHC molecule on DCs, we further investigated the link between IFN-γ and iNOS during EAE. We analyzed the influence of IFN-γ on iNOS-expression in astrocytes and CNS-infiltrated immune cells, and on apoptosis of the myelin-synthesizing cells, oligodendrocytes. For this purpose, we neutralized the IFN-γ with the anti-IFN-γ (100 μg/mouse; *i.v*.) mAb during MOG-induced active EAE. Consistent with several published reports (35, 36), our results showed exacerbation of the clinical symptoms of EAE with the neutralization of IFN-γ (Figure 5A). We also observed an increased infiltration of CD45^+^ leukocytes and CD4^+^ T cells in the brain as well as spinal cord (Figure 5B). Both the meninges and parenchyma showed increased infiltration of immune cells in the anti-IFN-γ-treated group, as compared to the isotype control IgG-treated mice (data not shown). While anti-IFN-γ-treated mice showed significantly low infiltration of CD11b^+^F4/80^+^GR-1^−^ cells, however, the numbers of CD11b^+^F4/80^−^GR-1^+^ cells were dramatically increased in the brain and spinal cord, as compared to control group (Figure 5C). The neutralization of IFN-γ using mAb did not induce iNOS expression in the astrocytes (Figure 5D). However, with anti-IFN-γ-treatment, iNOS expression was significantly induced in CD45^+^ leukocytes in both brain and spinal cord, as compared to isotype IgG-treated control mice (Figure 5D), suggesting that neutralization of IFN-γ leads to the reappearance of iNOS-expression on CD45^+^ leukocytes and possibly in microglia, similar to the effects observed with L-NAME treatment.

We then asked whether increased infiltration of CD11b^+^F4/80^−^GR-1^+^ cells could have an effect on the survival of oligodendrocytes during EAE. Our results showed that during EAE, the cleaved-caspase 3 signal co-localizes with CNPase^+^ oligodendrocytes, suggesting that they are undergoing apoptosis (Figure 5E). The anti-IFN-γ-treated mice showed significantly higher co-localization of cleaved-caspase 3 with CNPase^+^ oligodendrocytes in the brain, as compared to the isotype control group (Figure 5E), suggesting that the neutralization of IFN-γ signaling lead to increased apoptosis of oligodendrocytes during EAE. Furthermore, treatment with anti-IFN-γ mAb increased the infiltration of effector CD4^+^ T cells, CD11b^+^F4/80^−^GR-1^+^ cells, and iNOS-expressing inflammatory immune cells, and reduced infiltration of suppressive CD11b^+^F4/80^+^ cells (includes F4/80-expressing suppressive Mo-MDSCs) in the CNS. These could have contributed to increased oligodendrocyte and neuronal damage, consequently exacerbating the EAE pathology.

## Discussion

In the present study, we show that iNOS critically regulates neuroinflammation at different phases of EAE by controlling the infiltration of immune cells in the brain and spinal cord. Inhibition of iNOS in the antigen-priming phase or effector phase of EAE exacerbates the symptoms of the disease via selectively increasing the infiltration of inflammatory CD11b^+^F4/80^−^GR-1^+^ cells, while at the same time reducing the infiltration of CD11b^+^F4/80^+^GR-1^−^ cells in the brain and spinal cord, respectively. Similarly, with anti-IFN-γ mAb treatment, we showed differential infiltration of CD11b^+^F4/80^+^GR-1^−^ and CD11b^+^F4/80^−^GR-1^+^ cells in the CNS during EAE. Furthermore, lack of iNOS or neutralization of IFN-γ promoted the infiltration of CD11b^+^F4/80^−^GR-1^+^ cells in the brain or spinal cord and enhanced the inflammation and apoptosis of CNPase^+^ oligodendrocytes.

Compelling evidence has suggested that various types of myeloid cells do infiltrate into inflamed CNS along with myelin-reactive Th1 and Th17 cells (40). These cells contribute to the various inflammatory pathways leading to demyelination and axonal damage in the CNS. iNOS controls the function of a variety of myeloid and lymphoid cell populations in both a cell-intrinsic and extrinsic manner (41). iNOS is also critically involved in the pathogenesis of EAE and MS (17). However, how it affects neuroinflammation in the various phases of EAE was not clear. In the present work, we showed that inhibition of iNOS during the antigen-priming phase leads to a selectively increased infiltration of CD45^+^ leukocytes and CD4^+^ T cells selectively in the brain but not in the spinal cord. Together with data from iNOS^−/−^ mice, our results suggest that a lack of iNOS in the priming phase induces inflammation in the secondary lymphoid organs in a manner that causes pathogenic cells to be mobilized into the brain. The molecular details of trafficking of these cells across the blood-brain barrier to CNS and the effect of iNOS and possibly other isoform of NOS, such as eNOS in their transmigration need further investigation. Previous studies have shown that while iNOS does not affect the differentiation of Th1, Th2 and Tregs, it does influence Th17 differentiation (31). However, studies on the mechanism through which iNOS and NO influence Th17 differentiation have yielded mixed results. Studies in mice have revealed that this inhibitory function of endogenous iNOS is via the nitration of tyrosine residues in RORγt, and the inhibition of aryl hydrocarbon receptor (Ahr)-signaling (31, 42). However, others have reported that iNOS and NO support human Th17 differentiation via the cyclic guanosine monophosphate (cGMP)-dependent protein kinase pathway, and endogenous iNOS play an essential role in the stability of human Th17 cells when differentiated in the presence of IL-1β, IL-6, and IL-23 (43). Our data show that inhibition of NOS or genetic deficiency of iNOS neither affect the generation of pathogenic Th1, Th17 cells, and Foxp3^+^ regulatory CD4^+^ T cells nor IFN-γ and IL-17A-expressing γδ T cells in the secondary lymphoid organs.

iNOS regulates the generation and function of regulatory DCs that control the effector function of CD4^+^ and CD8^+^ T cells, and helps in amelioration of EAE (23–25). CD11b^+^F4/80^+^ cells and F4/80-expressing Mo-MDSCs are known to have a regulatory role in EAE (38). We show that a lack of iNOS function in the priming-phase results in a significant reduction in the frequency of CD11b^+^F4/80^+^ cells as well as F4/80-expressing Mo-MDSCs in the spleen. L-NAME-treatment or lack of iNOS in mice yielded significantly lower infiltration of these cells in the inflamed CNS. In contrast, lack of iNOS significantly increased the previously reported pathogenic CD11b^+^F4/80^−^GR-1^+^ cells in the brain (39). In contrast, inhibition of iNOS in the effector phase of EAE, when ongoing inflammation in the CNS causes demyelination and axonal damage, resulted in higher infiltration of CD4^+^ T cells and CD11b^+^F4/80^−^GR-1^+^ cells preferentially in the spinal cord, but not in the brain. These results suggest that temporal inhibition of iNOS during EAE modulates differential clinical pathology in the brain and spinal cord. iNOS inhibition during the antigen-priming phase of EAE showed a high frequency of iNOS-expressing CD45^+^ leukocytes selectively in the brain, but not in the spinal cord. Since CNS-resident microglia express iNOS under inflammation and microglia are also characterized by CD45^int^ expression, our study cannot exclude the potential role of iNOS-expressing microglia in exacerbation of EAE symptoms. Instead, this further opens the avenue to investigate the relative functions of iNOS-expressing microglia and circulation-derived macrophages in the early and late phases of neuroinflammation during EAE. The GFAP^+^ astrocytes are also an important source of iNOS-expression during neuroinflammation (16). We also observed higher iNOS-expressing astrocytes in the brain and spinal cord in EAE mice. However, inhibition of iNOS in the priming phase selectively reduced the frequency of iNOS-expressing GFAP^+^ astrocytes in the brain, whereas it was mostly unaffected upon inhibition of iNOS during the effector phase.

IFN-γ is the upstream regulator of iNOS expression in a variety of myeloid and lymphoid cells, and cells of non-hematopoietic lineages (9, 12). The neutralization of IFN-γ using anti-IFN-γ mAb during EAE in mice have been observed to result in more severe symptoms and pathology (44). IFN-γ is a known suppressor of Groα/KC (CXCL1) (45) and MIP-2 (CXCL2) (46), both being neutrophil chemoattractants that recruit neutrophils to the site of inflammation (47). In the absence of IFN-γ signaling, Th17 cells predominate infiltrate into the CNS, and predominantly recruit neutrophils in the CNS (48, 49), possibly via CXCL1- and CXCL2-mediated neutrophil chemoattraction. Neutralization of IFN-γ therefore promotes neutrophil trafficking to the inflamed CNS. Consequently, we reasoned that a lack of iNOS function during anti-IFN-γ treatment (12) may contribute for bias differentiation of CD11b^+^F4/80^+^ cells and F4/80-expressing Mo-MDSCs in the lymphoid organs, and infiltration of these cells in CNS may cause severe EAE. However, we also observed an increase in the expression of iNOS in the CNS-infiltrating immune cells and possibly CD45^int^ microglia in anti-IFN-γ-treated mice, suggesting that in the absence of IFN-γ, some other factors can also induce the expression of iNOS in the inflamed CNS (50). Furthermore, the enhanced expression of iNOS in the CNS infiltrating immune cells was associated with increased apoptosis of myelin-synthesizing oligodendrocytes in anti-IFN-γ-treated mice, suggesting that high level of iNOS produced by inflammatory CNS infiltrates can affect the remyelination process and contribute to severity of the disease. A study with cuprizone-induced demyelination model reported that infiltration and the inflammatory function of CXCR2^+^ neutrophils are required to induce oligodendrocyte damage and demyelination in addition to the toxic effect of cuprizone on mitochondrial function (51). The increased apoptosis of CNPase^+^ oligodendrocytes in anti-IFN-γ treated mice, might be in part due to increased neutrophilic infiltration in the CNS. A similar pathological mechanism may exist in iNOS-deficient, or L-NAME treated wild-type mice and warrants further investigation. We showed that MOG-induced inflammation caused the induction of iNOS-expression in the astrocytes, and that anti-IFN-γ treatment completely inhibits the iNOS expression in astrocytes. The numbers of iNOS-expressing astrocytes and the iNOS produced by them have different consequences. While low levels of iNOS or NO are beneficial, and their sustained high levels are detrimental to the CNS homeostasis (52). Therefore, the high levels of iNOS produced in the anti-IFN-γ recipients in our studies might have caused apoptosis of oligodendrocytes in the brain, and thus contributed to increased the clinical severity of the EAE. In conclusion, we have shown here that immunoregulatory role of iNOS safeguards the brain and spinal cord from inflammatory granulocytic infiltration during the antigen-priming and effector phase of EAE, respectively.

## Materials and Methods

### Mice

Wild-type C57BL/6 and iNOS^−/−^ (B6.129P2-*Nos2*^*tm*1*Lau*^/J) mice were obtained from The Jackson Laboratory (Bar Harbor, ME). Mice were maintained and bred in the Experimental Animal Facility of the National Centre for Cell Science (NCCS), Pune, India. All mice experiments were performed with the protocols approved by the Institutional Animal Ethics Committee. (Project Id: EAF/2016/B-257).

### Antibodies and Reagents

Alexa Fluor 488-CD4 (GK1.5), APC-eFluor 780-CD4 (GK1.5), FITC-γδTCR (GL3), APC-γδTCR (GL3), APC-CD45 (30F-11), FITC-F4/80 (BM8), APC-Cy7-F4/80 (BM8), FITC-Ly6G (1A8), Alexa Fluor 647-Ly6C (HK1.4), biotin-CD11b (M1/70), APC-GR-1 (RB6-8C5), PE-GM-CSF (MP1-22E9), Brilliant violet 421-IL-17A (TC11-18H10.1), and purified anti-mouse GFAP (MCA-5C10) were purchased from BioLegend (San Diego, CA). Biotin-CD11c (N418), PE/Cy7-IFN-γ (XMG1.2), Pacific blue-Foxp3 (FJK-16s), PE-Foxp3 (MF-14), PE-Eomes (Dan11mag), eFluor 450-IA^b^ (AF6-1201) were obtained from eBioscience (San Diego, CA). Purified anti-IFN-γ (XMG1.2), and rat IgG2b, k isotype control (LTF-2) were purchased from BioXcell (West Lebanon, NH). PE-Cy7-CD11b (M1/70) antibody was from BD Biosciences (San Diego, CA). Purified anti-iNOS antibody (EPR16635) was purchased from Abcam (Cambridge, MA). Purified cleaved caspase 3 (5A1E) and purified anti-CNPase (D83E10) was obtained from Cell Signaling Technology (Danvers, MA). *N-*ω-nitro-l-arginine methyl ester (L-NAME) was purchased from MP Biomedicals (Santa Ana, CA).

### Induction of Active EAE

Wild-type C57BL/6 or iNOS^−/−^ mice were given subcutaneous (*s.c*.) injections of an emulsion of MOG_35−55_ (MOG) (200 μg/mouse) in complete Freund's adjuvant (CFA) containing *Mycobacterium tuberculosis H37Ra* (5 mg/ml), and also given intravenous injections (*i.v*.) of pertussis toxin (PTx, 200 ng/mouse) at day 0 and 2. L-NAME was administered intraperitoneally (i.p. 100 mg/kg) daily from day 0 to 7 (priming phase) or day 8 to 15 (effector phase). Control animals received *i.p*. injections of PBS. For the neutralization of IFN-γ, animals were given anti-mouse IFN-γ mAb (clone XMG1.2; 100 μg/mouse) *i.v*. on days 0, 4, 8, 12, and 16. The animals that received isotype control IgG were used as a controls. Mice were followed for the development of clinical signs of EAE. Scoring of clinical symptoms was performed as follows; score 0, no symptoms; 1, limp tail or hind limb weakness but not both; 2, limp tail and hind limb weakness; 3, partial hind limb paralysis; 4, complete hind limb paralysis; and 5, death by EAE.

### Immunofluorescence Staining of the Brain and Spinal Cord

Mice were sacrificed, ice-cold PBS was transcardially perfused, and the brain and spinal cord were harvested. The tissues were immediately snap-frozen in OCT compound (Sakura Finetek, Torrance, CA). Seven-micrometer-thick cryosections were prepared using a cryomicrotome. Sections were fixed with chilled acetone for 5 min, air dried, followed by washing with PBS. The tissue sections were blocked with 10% horse serum (Jackson ImmunoResearch, West Grove, PA) in PBS at room temperature (RT) for 30 min, followed by washing thrice with ice-cold PBS. The sections were incubated with primary antibodies overnight (12–14 h) at 4°C, washed, further incubated with secondary antibodies at RT for 60 min, and then washed five times with PBS. The sections were stained with nuclear stain DAPI at RT for 5 min and washed twice with ice-cold PBS. Sections were mounted in mounting medium (Electron Microscopy Sciences, Hatfield, PA). Images were acquired on a Leica DMI6000 inverted fluorescent microscope (Leica Microsystems, Germany) at 100, 400, and 630x magnifications. Images were analyzed using Leica MMAF (Leica) or Image J software (National Institute of Health, Bethesda, MD).

The quantification of cells from microscopic images was performed using the MMAF software (Leica Microsystems, Germany). The single channel and multi-channel overlay images of sufficient magnification (original magnification, 400x) were used for quantification of the number of cells. At least 10 different microscopic fields per organ and more than three mice/group were analyzed. Each cell was analyzed for its cellular morphology, nuclear staining with DAPI, and specific markers. For evaluating cellular infiltration into the CNS, complete brain tissues sections were used.

### Flow Cytometry

Cells were harvested from spleen and draining lymph nodes of the mice. RBCs were lysed using ACK lysis buffer, and single cell suspensions were prepared. Cells were surface-stained with PE/Cy7-anti-mouse CD11b, PE/Cy5-anti-mouse CD11c, Alexa fluor 647-anti-mouse Ly6C, FITC-anti-mouse Ly6G, APC/Cy7-anti-mouse F4/80 and pacific blue-anti-mouse I-Ab (MHC class II) antibodies. Cells were incubated on ice in the dark for 30 min, washed with ice-cold PBS, and fixed with 1% paraformaldehyde. Cells were acquired on FACS Canto-II (BD Biosciences), and data were analyzed using the FlowJo software.

### Intracellular Cytokine Staining

Single cell suspensions were prepared from spleen and draining lymph nodes of mice. For transcription factor analysis, 1 × 10^6^ cells were stained for the surface molecules CD4 and γδ TCR on ice for 30 min and washed with ice-cold PBS. Cells were subjected to fixation, and permeabilization with the Foxp3 fixation/permeabilization buffer kit (Biolegend), and intracellular staining for Foxp3, T-bet, RORγt, and Eomes was performed as per the manufacturer's instructions. For intracellular cytokine analysis, cells (6 × 10^6^ cells/well) were stimulated with phorbol myristate acetate (PMA; 50 ng/ml) and ionomycin (850 ng/ml) in the presence of brefeldin-A (5 μg/ml) and monensin (2 μM) in 500 μl/well complete RPMI 1,640 medium in 24-well plates at 37°C in a humidified 5% CO_2_ incubator for 6 hours. Cells were collected, washed with PBS, and stained for the surface molecules CD4 and γδ TCR on ice for 30 min, and washed with ice-cold PBS. Cells were subjected to fixation and permeabilization using the Foxp3 fixation/permeabilization buffer kit (Biolegend) and intracellular staining for IL-17A, Foxp3, Eomes, and IFN-γ was performed as per the manufacturer's instructions. Cells were acquired on FACS Canto II (BD Bioscience), and data were analyzed using the FlowJo software.

### Statistical Analysis

Statistical comparisons were performed using the GraphPad Prism 6 software (GraphPad, San Diego, CA). Unpaired two-tailed Student's *t*-test was used to compare two variables. The ANOVA test was used to compare the means of more than two groups followed by appropriate multiple comparison tests. The other statistical methods used are described in the figure legends. A *p* < 0.05 was considered statistically significant.

## Ethics Statement

All mice experiments were performed with the approved protocols from the NCCS Institutional Animal Ethics Committee (Project Id: EAF/2016/B-257).

## Author Contributions

SAS and GL designed the experiments, analyzed the data, and wrote the manuscript. SAS performed all the experiments.

### Conflict of Interest Statement

The authors declare that the research was conducted in the absence of any commercial or financial relationships that could be construed as a potential conflict of interest.

## Supplementary Material

Figure S1 Inhibition of iNOS or lack of iNOS does not affect the generation of Th1, Th17, and Tregs in the secondary lymphoid organs. **(A)** EAE was induced in C57/BL/6 mice as shown in Figure 1A. The intracellular expression of IL-17A and IFN-γ in the γδ T cells of the spleen and draining lymph nodes of the mice was monitored and plotted (left). The dot plot shown is gated on γδ T cells. Quantifications of the percentage of intracellular cytokines in the γδ T cells are shown (right). **(B,C)** C57BL/6 and iNOS^−/−^mice were *s.c*. injected with 200 μg MOG_35−55_ (MOG) in CFA emulsion, and two doses of *i.v*. pertussis toxin (PTx, 200 ng/mouse) at day 0 and 2. Mice were administered *i.p*. L-NAME from day 0 to 7 daily. Control groups were given PBS. On the day 8, single cell suspensions were prepared from spleen and draining lymph nodes, and expression of the transcription factors, Eomes, Foxp3, RORγt, and T-bet in CD4^+^ T cells from the **(B)** spleen and **(C)** draining lymph nodes were analyzed using flow cytometry. Dot plots show the expression of the indicated transcription factors in CD4^+^ T cells. Numbers in the dot plots show percentage of indicated molecules in γδ T cells **(A)** and CD4^+^ T cells **(B,C)**. The horizontal line denotes mean and error bars represents ± SEM **(A)**. Student's *t*-test **(A)**. *n* = 4 mice/group **(A)** and 5 mice/group **(B)**.

Figure S2 Inhibition of iNOS or its deficiency differentially regulates the infiltration of myeloid cells in the CNS. The brain and spinal cord tissue sections of mice from Figures 3A,E were stained with CD11b (red), F4/80 (green), GR-1 (light blue) and nuclear stain DAPI (dark blue). **(A)** Representative images of the brain and spinal cord of mice either untreated or treated with L-NAME in the effector phase of EAE are shown (upper). Magnified views of the areas marked with the dotted squares are shown next to the images. The mean number of infiltrated CD11b^+^F4/80^−^GR-1^−^, CD11b^+^F4/80^+^GR-1^−^ and CD11b^+^F4/80^−^GR-1^+^ cells from at least 11–14 fields of the brain and spinal cord sections were quantitated and shown (lower). **(B)** Representative images of the brain and spinal cord sections of wild-type and iNOS^−/−^ mice with EAE at day 20 are shown (upper). Magnified views of the areas marked with the dotted squares are shown next to the images. The mean numbers of infiltrated CD11b^+^F4/80^−^GR-1^−^, CD11b^+^F4/80^+^GR-1^−^ and CD11b^+^F4/80^−^GR-1^+^ cells from at least 19–29 fields of the brain and spinal cord were quantitated and shown (lower). Original magnification, 400x **(A,B)**. Scale bar, 100 μm **(A,B)**. ^*^*p* < 0.05, ^**^*p* < 0.01, ^***^*p* < 0.001, ^****^*p* < 0.0001. Student's *t*-test **(A,B)**. Error bars represents ± SEM **(A,B)**. *n* = 5 mice/group.

## Abbreviations

APCs: Antigen-presenting cells
BMDCs: Bone marrow-derived DCs
CNPase, 2': 3'-cyclic nucleotide 3'-phosphodiesterase
CNS: Central nervous system
CFA: Complete Freund's adjuvant
DCs: Dendritic cells
EAE: Experimental autoimmune encephalomyelitis
eNOS: Endothelial NOS
GFAP: Glial fibrillary acidic protein
iNOS: inducible NOS
L-NAME: *N-*ω-nitro-l-arginine methyl ester
MDSCs: Myeloid-derived suppressor cells
MOG: Myelin oligodendrocyte glycoprotein 35-55 peptide
Mo-MDSCs: Monocytic myeloid-derived suppressor cells
MS: Multiple sclerosis
NO: Nitric oxide
NOS: Nitric oxide synthase
nNOS: neuronal NOS.
