# Supplementary figures and images for: The iNOS Activity During an Immune Response Controls the CNS Pathology in Experimental Autoimmune Encephalomyelitis
Source: Front Immunol. 2019 Apr 4;10:710. doi: 10.3389/fimmu.2019.00710 (PMC6458273; doi:10.3389/fimmu.2019.00710)

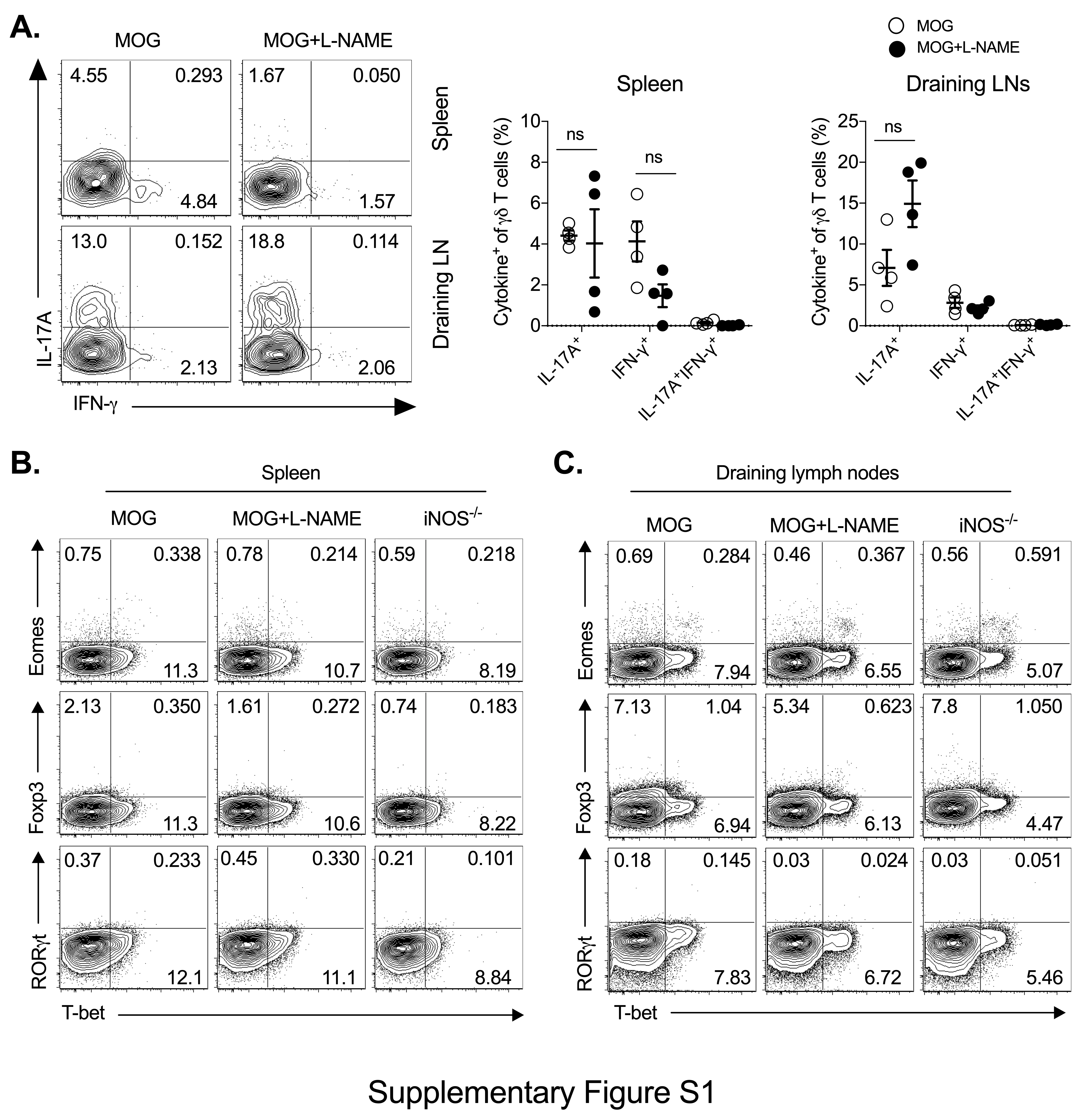

Supplement: Figure S1 — Inhibition of iNOS or lack of iNOS does not affect the generation of Th1, Th17, and Tregs in the secondary lymphoid organs. (A) EAE was induced in C57/BL/6 mice as shown in Figure 1A. The intracellular expression of IL-17A and IFN-γ in the γδ T cells of the spleen and draining lymph nodes of the mice was monitored and plotted (left). The dot plot shown is gated on γδ T cells. Quantifications of the percentage of intracellular cytokines in the γδ T cells are shown (right). (B,C) C57BL/6 and iNOS−/−mice were s.c. injected with 200 μg MOG35−55 (MOG) in CFA emulsion, and two doses of i.v. pertussis toxin (PTx, 200 ng/mouse) at day 0 and 2. Mice were administered i.p. L-NAME from day 0 to 7 daily. Control groups were given PBS. On the day 8, single cell suspensions were prepared from spleen and draining lymph nodes, and expression of the transcription factors, Eomes, Foxp3, RORγt, and T-bet in CD4+ T cells from the (B) spleen and (C) draining lymph nodes were analyzed using flow cytometry. Dot plots show the expression of the indicated transcription factors in CD4+ T cells. Numbers in the dot plots show percentage of indicated molecules in γδ T cells (A) and CD4+ T cells (B,C). The horizontal line denotes mean and error bars represents ± SEM (A). Student's t-test (A). n = 4 mice/group (A) and 5 mice/group (B). [file Image_1.TIFF]

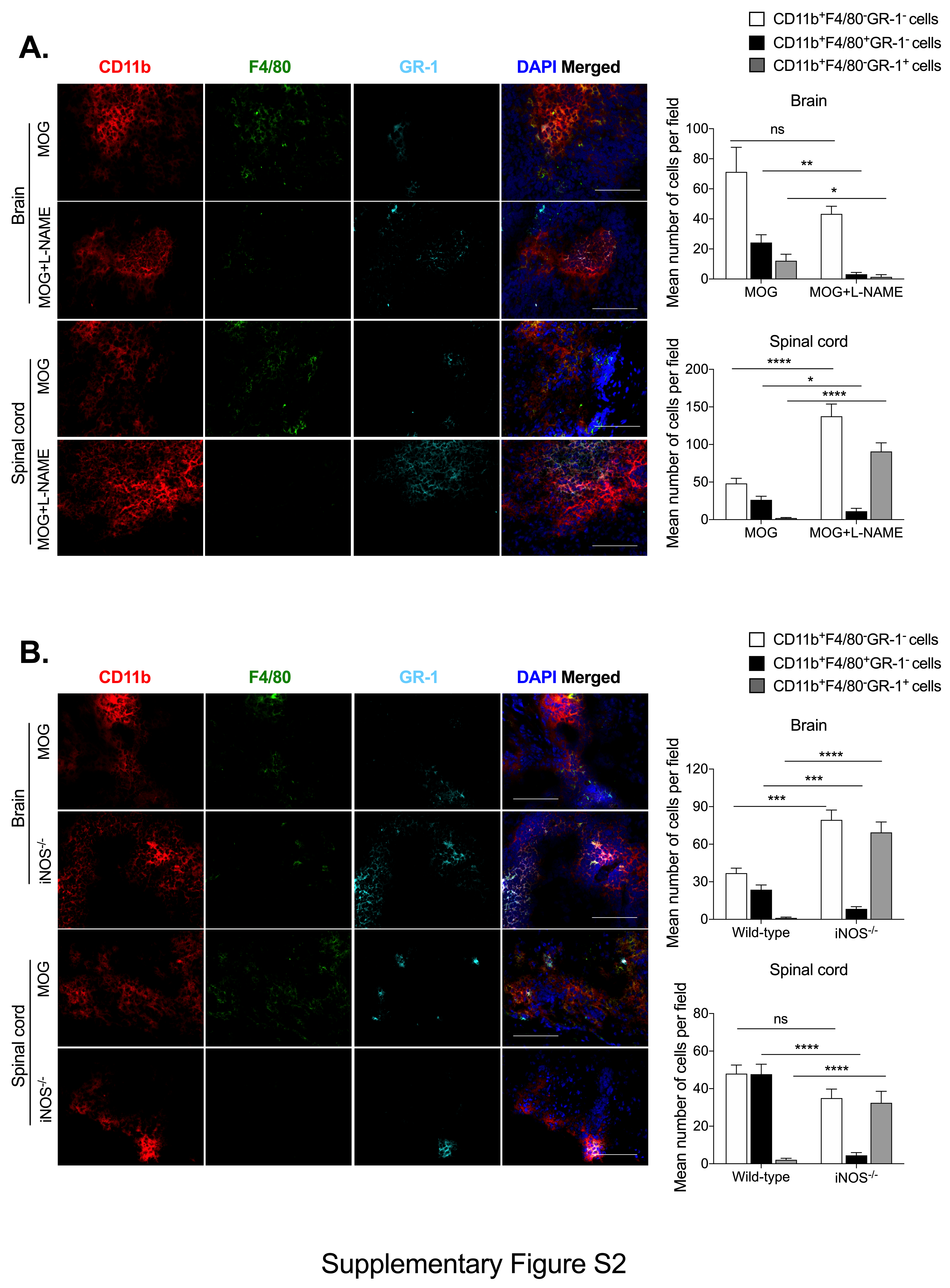

Supplement: Figure S2 — Inhibition of iNOS or its deficiency differentially regulates the infiltration of myeloid cells in the CNS. The brain and spinal cord tissue sections of mice from Figures 3A,E were stained with CD11b (red), F4/80 (green), GR-1 (light blue) and nuclear stain DAPI (dark blue). (A) Representative images of the brain and spinal cord of mice either untreated or treated with L-NAME in the effector phase of EAE are shown (upper). Magnified views of the areas marked with the dotted squares are shown next to the images. The mean number of infiltrated CD11b+F4/80−GR-1−, CD11b+F4/80+GR-1− and CD11b+F4/80−GR-1+ cells from at least 11–14 fields of the brain and spinal cord sections were quantitated and shown (lower). (B) Representative images of the brain and spinal cord sections of wild-type and iNOS−/− mice with EAE at day 20 are shown (upper). Magnified views of the areas marked with the dotted squares are shown next to the images. The mean numbers of infiltrated CD11b+F4/80−GR-1−, CD11b+F4/80+GR-1− and CD11b+F4/80−GR-1+ cells from at least 19–29 fields of the brain and spinal cord were quantitated and shown (lower). Original magnification, 400x (A,B). Scale bar, 100 μm (A,B). *p < 0.05, **p < 0.01, ***p < 0.001, ****p < 0.0001. Student's t-test (A,B). Error bars represents ± SEM (A,B). n = 5 mice/group. [file Image_2.TIFF]
